# Harmonizing epidemiological research methodology for atopic dermatitis research: protocol for the EPISTAR international consensus exercise

**DOI:** 10.1093/skinhd/vzaf047

**Published:** 2025-07-28

**Authors:** Suzanne H Keddie, Karl Philipp Drewitz, Katrina Abuabara, Sebastien Barbarot, Kelly Barta, Aaron M Drucker, Jinane El Khoury, Ousmane Faye, Cesar Galvan, Kiran Godse, Rita J Iskandar, Jennifer J Koplin, Tina Mesarič, Yukihiro Ohya, Erere Otrofanowei, Christian Vestergaard, Hua Wang, Hywel C Williams, Yik Weng Yew, Carsten Flohr, Christian J Apfelbacher

**Affiliations:** Global Atopic Dermatitis Atlas, St John’s Institute of Dermatology, St Thomas’ Hospital, Guy’s and St Thomas’ NHS Foundation Trust, London, UK; Institute of Social Medicine and Health Systems Research, Otto von Guericke University, Magdeburg, Germany; Department of Dermatology and Computational Precision Health, University of California San Francisco (UCSF), San Francisco, CA, USA; Department of Dermatology, Nantes Université, CHU Nantes, Nantes, France; Coalition of Skin Diseases, Washington DC, USA; Division of Dermatology, Department of Medicine, University of Toronto, Toronto, ON, Canada; Department of Medicine and Research and Innovation Institute, Women’s College Hospital, Toronto, ON, Canada; Dermatology Department, Gilbert and Rose Marie Chagoury School of Medicine, Lebanese American University, Beirut, Lebanon; Bamako Hospital of Dermatology, University of Sciences, Technics and Technology, Bamako, Mali; Emedic Salud, San Isidro, Lima, Peru; Clinica Internacional, Lima, Peru; Dr D Y Patil Medical College and Hospital, School of Medicine, Navi Mumbai, India; Department of Medicine and Research and Innovation Institute, Women’s College Hospital, Toronto, ON, Canada; Dermatology Department, Gilbert and Rose Marie Chagoury School of Medicine, Lebanese American University, Beirut, Lebanon; Leslie Dan Faculty of Pharmacy, University of Toronto, Toronto, ON, Canada; Child Health Research Centre, University of Queensland, Brisbane, QLD, Australia; Murdoch Children’s Research Institute, Melbourne, VIC, Australia; Institute Atopika, Maribor, Slovenia; Department of Occupational and Environmental Health, Graduate School of Medical Sciences, Nagoya City University, Nagoya, Japan; Division of General Allergy, Bantane Hospital, Fujita Health University, Allergy Centre, National Centre for Child Health and Development, Nagoya, Japan; Department of Medicine, College of Medicine University of Lagos and Lagos University Teaching Hospital, Lagos, Nigeria; Department of Dermatology, Aarhus University Hospital, Aarhus, Denmark; Department of Dermatology, National Clinical Research Center for Child Health and Disorders, Ministry of Education Key Laboratory of Child Development and Disorders, Children’s Hospital of Chongqing Medical University, Chongqing, China; Centre of Evidence-Based Dermatology, Lifespan and Population Health, University of Nottingham, Nottingham, UK; Lee Kong Chian School of Medicine, Nanyang Technological University, Singapore; Global Atopic Dermatitis Atlas, St John’s Institute of Dermatology, St Thomas’ Hospital, Guy’s and St Thomas’ NHS Foundation Trust, London, UK; Institute of Social Medicine and Health Systems Research, Otto von Guericke University, Magdeburg, Germany

## Abstract

**Background:**

Epidemiological studies of atopic dermatitis lack standardization in key areas, including how the burden is collected and reported, diagnostic criteria, sociodemographic factors and measurement of disease severity. Therefore, direct cross-study comparisons, recognition of population differences and pooled analyses are challenging or not possible. Consequently, the burden of atopic dermatitis remains difficult to assess and address. The Epidemiological Study Designs for Atopic Dermatitis Research (EPISTAR) initiative aims to reach consensus on which domain items should be recommended for future population-based epidemiological studies on atopic dermatitis and how they should be assessed.

**Methods:**

In phase 1, a steering group consisting of experts from dermatology and epidemiology as well as patient representatives will generate an initial list of items constituting key variables to measure. This list will be created by reviewing the existing literature, prioritizing evidence from systematic reviews where available. Phase 2 will include an international consensus exercise, conducted through eDelphi methodology. Phase 3 will involve an online consensus conference. In each Delphi round, international participants from diverse stakeholder groups will be invited to assess each item by rating their level of agreement with the item and methods by which it can be measured. Items that reach consensus will be removed after each round. Data analysis will follow predefined consensus criteria, with raw numbers, means and frequencies reported.

**Discussion:**

This harmonized approach has the potential to transform the field of atopic dermatitis epidemiology by addressing gaps in data quality and comparability, facilitating meta-analyses, and ultimately informing evidence-based policy and clinical guidelines.

## Teaser

The Epidemiological Study Designs for Atopic Dermatitis Research (EPISTAR) initiative aims to reach consensus on which domain items should be recommended for future population-based epidemiological studies on atopic dermatitis and how they should be assessed. This harmonized approach has the potential to transform the field of atopic dermatitis epidemiology by addressing gaps in data quality and comparability, facilitating meta-analyses, and ultimately informing evidence-based policy and clinical guidelines.

Atopic dermatitis is a common inflammatory skin condition,^1^ most notably recognized for its burden in young children, with an estimated prevalence of up to 20%, and 10% in adults.^2^ However, there is increasing recognition of the burden of atopic dermatitis throughout the lifespan, particularly in older adults,^3,4^ as well as the variations in clinical presentation across geographical regions^5^ and in different skin types.^6^ The significant impact of atopic dermatitis on patients’ lives is reflected in the global estimate of 123 disability-adjusted life years (DALYs) for atopic dermatitis in 2017 (95% confidence interval 66.8–205), this ranks atopic dermatitis fifteenth among all non fatal diseases and first among skin diseases globally.^7^ Despite being an important public health problem, substantial gaps remain in our understanding of the burden of atopic dermatitis in specific populations. A recent systematic review of atopic dermatitis prevalence data spanning 30 years^8^ found that over 40% of countries worldwide currently lack epidemiological data on atopic dermatitis, with disproportionate gaps in the African continent.^8^ Filling gaps in epidemiological data, including longitudinally, is crucial in understanding the global burden of disease and identifying key risk factors, to provide essential evidence to inform public health interventions (e.g. educational programmes) and policy decisions. As a result, there is a need for more epidemiological studies on atopic dermatitis globally.

Current epidemiological studies on atopic dermatitis are characterized by heterogeneity in nearly all aspects. Case definitions of atopic dermatitis are highly variable, sometimes using physician diagnosis and others relying on self-reports or criteria from participant questionnaires. The impact of the chosen case definition has been shown to significantly influence the prevalence or incidence estimates reported. For example, during a multi centre study in children, the mean centre prevalence of atopic dermatitis, when using a validated questionnaire was 9.4% vs. 3.9% when atopic dermatitis was defined by a skin examination from a physician.^9^ Additionally, the definition used will differ depending on whether prevalence or incidence is being measured^10^ and may differ in studies using routinely collected health data (e.g. using International Classification of Diseases codes) vs. primary research data (e.g. questionnaires delivered in a community).^11^

The International Study of Asthma and Allergies in Childhood (ISAAC) study was established to determine the burden of asthma, hay fever and eczema in paediatric populations worldwide. ISAAC used the same standardized methodology across all study centres, for instance with regard to the diagnosis and assessment of disease severity.^12^ It has since been widely implemented across 106 countries and 5 continents.^13^ While ISAAC has proven particularly valuable for epidemiological studies in settings lacking dermatologists for skin examinations, the questionnaire tools and skin examination protocol were validated and used in children aged 6–7 years and 13–14 years. The survey has since been used in other paediatric age groups, such as 1–5 year olds,^14^ but the validity of the ISAAC eczema questionnaire in infants and adults remains unclear. Additionally, the global understanding of disease severity remains limited, and more advanced methods for assessing severity have since been developed [e.g. in the Harmonizing Outcome Measures for Eczema (HOME) initiative^15^], which may be better suited for contemporary observational epidemiology studies. While ISAAC remains a key component of case ascertainment and will be included in this consensus exercise, additional items would benefit from a broader consensus process that encompasses all burden studies on atopic dermatitis.

Additional sources of variability include the prevalence measures reported (e.g. point, period or lifetime),^9,16,17^ the sociodemographic data collected (which can result in stratification of results by non aligning age groups), and whether or how disease severity and quality of life are also reported. As such, a greater number of epidemiological studies alone are not sufficient to fill knowledge gaps on the burden of atopic dermatitis. Future population-based studies on atopic dermatitis should ideally adopt a harmonized design to ensure that results capture key demographic, lifestyle and disease-related factors for stratification, and accurately report study methodologies and associated comorbidities, ultimately allowing for better comparability.

- The Epidemiological Study Designs for Atopic Dermatitis Research (EPISTAR) consensus exercise aims to reach consensus on which domain items should be recommended for future population-based epidemiological studies (­cross-sectional and cohort) on atopic dermatitis and how they should be assessed, including case ascertainment, disease severity, associated diseases and basic sociodemographic factors.

EPISTAR is part of the Global Atopic Dermatitis Atlas (GADA) project, the international initiative to create and maintain a resource for all data on atopic dermatitis worldwide.^18^

## Materials and methods

### Overview of the approach and timeline

**Figure 1:**
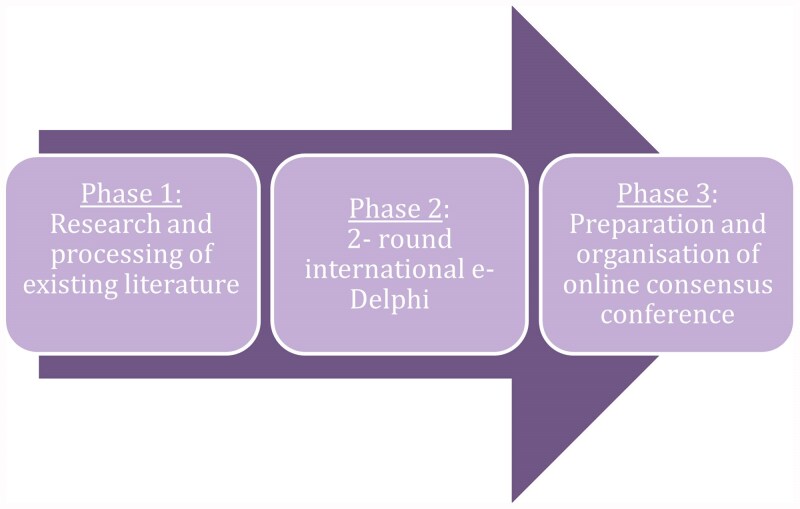
Projectflow and anticipated Delphi rounds for soliciting opinion on the recommended set of domains and domain items for inclusion in future epidemiology studies of atopic dermatitis.

A steering group will be formed with the goal of ensuring representation from all continents as fully as possible. A modified Delphi approach involving participants from relevant stakeholder groups will be employed, integrating multiple Delphi rounds, collaboration with an expert steering group and a concluding online Consensus Conference meeting. The timeline for Delphi rounds and the final consensus conference are depicted in Figure 1. This streamlined consensus process draws extensively on prior consensus exercises, systematic reviews and large-scale population-based atopic dermatitis studies, leveraging the expertise of the Study Management and Steering Groups. The study’s outcomes will be reported in adherence to the Accurate Consensus Reporting Document (ACCORD) guidelines.^19^

### Key assumptions and definitions

#### Study designs

The set of recommended items developed will cover population-based studies of atopic dermatitis, of cross-sectional and cohort design, measuring either disease prevalence or incidence.

#### Consensus

The field of atopic dermatitis is not new to consensus methodology. Consensus exercises have been used to Harmonize Outcomes Measures for Eczema (HOME) in trials,^20^ as well as in the international Treatment of Atopic eczema (TREAT) Registry Taskforce to find consensus on core domains and domain items for atopic dermatitis registers,^21^ and in the production of guidelines to support patients and clinicians in the optimal treatment of atopic dermatitis.^22^ The employed consensus methods are designed to facilitate group decision-making, combining existing evidence to reach a collective agreement, especially when there is a lack of scientific evidence or where there exists contradictory evidence.^23^ The Delphi method is a structured, iterative process that gathers expert opinions through multiple rounds of anonymous feedback and controlled discussions, aiming to converge on a consensus without direct interaction.^24^

Throughout this consensus exercise (including Steering Group meetings, the eDelphi and the final consensus conference), achievement of consensus will be defined as when fewer than 30% of participants disagree and at least 70% of participants agree. Conversely, lack of consensus will be defined as when 30% or more of participants disagree, or when agreement does not reach at least 70%. These thresholds have been used before in similar international projects.^25^

### Organization and procedure

#### Role of the steering group

In the first phase, the EPISTAR Steering Group (ESG) will generate the ‘long list’ of domains and items to be voted on. In consensus methodology, the ‘long list’ refers to an initial collection of domains and items that are considered relevant to the research topic. Domains represent broad conceptual categories, while items define the specific variables within each domain. Another important responsibility of the ESG is the scientific oversight of the Delphi process and consensus meetings. A Study Management Group (SMG) will be formed consisting of C.J.A., C.F., S.H.K. and K.P.D., who are responsible for the operational and managerial tasks of the consensus exercise. The ESG will include members from all five continents, bringing together dermatologists, clinical epidemiologists, methodologists and patient representatives. Steering group members will be approached and selected based on their expertise in these key areas, as well as their availability, geographical representativeness, and a balance of male and female members to ensure diverse perspectives. Details of the steering group members and their participation will be included in the published consensus findings.

#### Delphi participants

Participants invited to join this international consensus exercise are expected to possess expertise in atopic dermatitis and have experience in epidemiology. To ensure that the recommendations are globally applicable, the recruitment strategy prioritizes representation from diverse countries and regions. Participants will be selected from the following stakeholder groups: epidemiologists, methodologists, clinicians, patients and policymakers. Specific individuals within each group are identified through the GADA networks, such as the International League of Dermatological Societies (ILDS), the International Eczema Council, the International Alliance of Dermatology Patient Organizations (GlobalSkin) and national dermatology societies. Additional outreach is conducted through connections made at international conferences, including the International Society of Atopic Dermatitis and the European Academy of Dermatology and Venereology. The steering group members will also leverage their personal and professional networks to enhance recruitment efforts.

The geographical distribution of Delphi participants is essential to this project. To address this, we propose reviewing respondent locations midway through round 1 of the Delphi. Participants will be asked to provide their location at the start of the survey, but no additional data will be collected at this stage. This approach will enable us to refine our outreach strategy and target advertising in under-­represented regions.

#### Identification of domain items

Domains and items for consensus, as well as how these items should be measured, were identified through a review of the existing literature, including systematic reviews, guidelines and previous consensus exercises. Previous consensus exercises in atopic dermatitis have identified core outcome sets for atopic dermatitis registers^26^ and atopic dermatitis clinical trials.^15^

Key literature identified in highlighting these items and domains are listed below.

- The ISAAC study developed a questionnaire for identifying symptoms of eczema in children, which is widely used across 106 countries.^13^ This questionnaire also includes key demographic questions and was modelled from the UK refinement of the Hanifin and Rajka diagnostic criteria for atopic dermatitis.
- The HOME initiative recommended four core domains that should be measured in all eczema trials: clinical-reported signs, patient-reported symptoms, quality of life and long-term control.^15^
- The TREAT register taskforce defined a core dataset for registers of paediatric and adult patients with atopic dermatitis on phototherapy or systemic therapy.^26^

For EPISTAR, the items identified have been categorized in two key domains – distribution and determinants – aligned with the foundational definition of epidemiology by Lilienfeld and Stolley in 1980:^27^

‘Epidemiology is concerned with the patterns of disease occurrence [distribution] in human populations and the factors that influence these patterns [determinants].’

These two domains represent the core pillars of epidemiology and form the basis of this consensus exercise. A list of the items under consideration in each domain is provided in Table 1. In this consensus exercise, we aim to reach agreement only on items related to case ascertainment, severity, comorbidities and basic sociodemographic factors. Additional resources will be provided on other important confounding factors that researchers may wish to consider, such as lifestyle, environmental and genetic factors, but these will not be included in the present consensus exercise.

**Table 1:** Items so far identified by the steering group as criteria to be considered in future epidemiological studies of atopic dermatitis, split into two domain categories: distribution and determinants

Distribution	Determinants
Case ascertainment (mode of diagnosis, diagnostic criteria, severity)	Time (e.g. season) Age Sex Country of residence Ethnicity Socioeconomic position Comorbidities (personal and family)

#### Modified eDelphi and approach for soliciting consensus in each round

To address the lack of consensus on which items should be collected in every observational, population-based study of atopic dermatitis and how these items should be assessed, a modified eDelphi method was chosen. This approach incorporates both an initial Steering Group to collate and propose items for a Delphi exercise and an online consensus conference meeting to review, confirm and validate the results of the Delphi exercise. The Delphi method is widely recognized in health research for its effectiveness in achieving consensus.^28–30^

A key advantage of this method, and a primary reason for its selection, is its suitability for online implementation, ensuring global representation while avoiding the logistical challenges of in-person meetings. The anonymity afforded by the Delphi process reduces the potential influence of group dynamics or peer pressure, fostering unbiased opinions.^23^

The exercise will include two iterative Delphi rounds. International recommendations suggest conducting between two and five Delphi rounds,^31^ with a systematic review of 100 studies employing the Delphi method finding two rounds to be the most common.^31^ Limiting the process to two rounds minimizes participant dropout and helps maintain a high response rate throughout the exercise.

Prior to the start of each round, a 2-week testing period will be conducted to confirm the software functionality and address any potential interpretation challenges with the proposed questions. The first Delphi round of questions on which items should be measured will be developed based on a review of existing literature conducted by the SMG.

Round 1 of the Delphi will ask participants how important each item brought for consensus by ESG is in inclusion of a recommended set of items to be included in all population-based epidemiology studies of atopic dermatitis. Additionally, participants will work to reach consensus on how each item should be measured. Each question will be assessed using a 5-point Likert scale, ranging from 1 (strongly agree) to 5 (strongly disagree), with intermediate options for agree, neither agree nor disagree, and disagree. Where 1–2 (strongly agree and agree) will be categorized as ‘agreement for’ and 4–5 (disagree and strongly disagree) will be categorized as ‘agreement against’. A 5-point Likert scale was specifically chosen because, in a recent comparison with a 9-point scale, it led to twice as many outcomes reaching consensus.^32^ Given that this study is designed to include only two rounds of Delphi, maximizing consensus is particularly important. A free-text field will also be included for participants to provide any additional comments or suggest additional items to be included.

Results from round 1 will be collated, any items achieving consensus of ‘agreement against’ will be removed from the questions for round 2. Similarly, those achieving consensus of ‘agreement for’ will also no longer be brought into the second round but will only be recalled for confirmation at the consensus conference. Anonymized results from round 1 will be shared with all participants prior to the launch of round 2 and during round 2, participants will be able to see their scores of the previous round. Any items that do not reach consensus in round 2 will be brought for discussion at the final consensus conference meeting.

The modified eDelphi will be administered through the Qualtrics XM Platform (Qualtrics, Seattle, WA, USA). Qualtrics is an online survey platform that has been widely implemented in consensus studies.^33,34^ The Delphi survey will be implemented in English with an introductory statement explaining the survey in each of the top 10 most commonly spoken languages.

A final online consensus conference will be scheduled to discuss any items that do not reach consensus in round 1 or round 2, and to confirm the final list of items to be included in the recommendation.

### Ethics and consent

We consulted the UK National Health Service Health Research Authority research tool with the conclusion that we do not need to seek ethical review for this study. All personalized data (limited to a necessary minimum: age, sex at birth, resident World Health Organization region, stakeholder group) will be available to the study investigators, but data shared with panellists or published will be aggregated. All participants will be required to agree to participate and consent to this use of their data.

### Evaluation and analyses

At the end of each round, we will summarize the results, presenting absolute numbers and percentages across all participants and by stakeholder group. Checking for non-­response bias via the Mann–Whitney *U* test was encouraged in a recent review of Delphi studies.^35^ This analysis and all other descriptive statistics will be carried out using R statistical software (v4.4.1).^36^

Results of the initial research will be presented, along with the findings from the eDelphi and consensus conference. As the main result, we will report all domains items that reached consensus ‘in’ or consensus ‘out’ criteria, respectively. Participant characteristics and notable differences between stakeholder preferences will also be reported.

## Discussion

The EPISTAR consensus exercise aims to establish a recommendation of a minimum set of domain items to be collected in future population-based epidemiological studies on atopic dermatitis and to determine the most appropriate methodologies for their assessment. By promoting harmonization across epidemiological research, this consensus exercise addresses the critical need for standardization, ensuring findings from individual studies are comparable and collectively enhance our understanding of the global burden of atopic dermatitis.

While the potential benefit of this study is clear, there are some limitations to this study design. The global applicability of this study is reliant on the number of participants from a wide range of countries and all stakeholder groups. While we can actively encourage participation, we cannot ultimately control the number of participants from each country and stakeholder group. Further, while carrying out a two-round eDelphi allows for quicker consensus there is the chance that we lose some of the nuances that can be gained from implementing a Delphi with a greater number of rounds.

The output of recommended domain items is intended to provide consistency while retaining flexibility for researchers to include additional measures tailored to their specific research questions. Similar to the adoption of core outcome sets in clinical trials, the recommended domains identified through EPISTAR are not intended to restrict the scope of research but to provide a foundational framework for interoperability and data integration across studies.

To support implementation of these recommendations, we plan to engage with key stakeholders early and disseminate the findings widely through academic publications, presentations and professional networks. By publishing and publicizing these recommendations, the exercise seeks to encourage widespread adoption, fostering a collaborative and standardized approach to understanding the epidemiology of atopic dermatitis on a global scale.

## Data Availability

The data underlying this article are available in the article.
